# A Novel Blind Separation Method in Magnetic Resonance Images

**DOI:** 10.1155/2014/726712

**Published:** 2014-02-23

**Authors:** Jianbin Gao, Qi Xia, Lixue Yin, Ji Zhou, Li Du, Yunfeng Fan

**Affiliations:** ^1^School of Resources and Environment, University of Electronic Science and Technology of China, Chengdu 611731, China; ^2^Key Laboratory of Integrated Electronic System, Ministry of Education, Chengdu 611731, China; ^3^Sichuan Academy of Medical Sciences & Sichuan Provincial People's Hospital, Chengdu 610072, China; ^4^School of Computer Science & Engineering, University of Electronic Science and Technology of China, Chengdu 611731, China; ^5^Southwest China Research Institute of Electronic Equipment, Chengdu 610036, China

## Abstract

A novel global search algorithm based method is proposed to separate MR images blindly in this paper. The key point of the method is the formulation of the new matrix which forms a generalized permutation of the original mixing matrix. Since the lowest entropy is closely associated with the smooth degree of source images, blind image separation can be formulated to an entropy minimization problem by using the property that most of neighbor pixels are smooth. A new dataset can be obtained by multiplying the mixed matrix by the inverse of the new matrix. Thus, the search technique is used to searching for the lowest entropy values of the new data. Accordingly, the separation weight vector associated with the lowest entropy values can be obtained. Compared with the conventional independent component analysis (ICA), the original signals in the proposed algorithm are not required to be independent. Simulation results on MR images are employed to further show the advantages of the proposed method.

## 1. Introduction

Blind source separation (BSS) aims at recovering unknown source signals only from the observed data. It has received considerable attention for its potential applications in a lot of fields, such as biomedical signal processing, image processing, and digital communications. The basic instantaneous linear mixture model used in BSS is as follows:

(1) $$\begin{array}{c} x\left(t\right)=As\left(t\right), \end{array}$$

where *s*(*t*) = [*s*_1_(*t*), *s*_2_(*t*),…*s*_*m*_(*t*)]^*T*^ is a *m* × 1 vector of source signals which represents the samples of unobserved source signals, *x*(*t*) = [*x*_1_(*t*), *x*_2_(*t*),…, *x*_*n*_(*t*)]^*T*^ is a *n* × 1 vector of mixed signals observed by *n* sensors, and *A* = [*a*_1_, *a*_2_,…, *a*_*m*_] is an unknown *n* × *m* mixing matrix of full rank. Assume the weight matrix *W* ∈ *R* ^*n*×*m*^ and the output $\hat{s}(t)=Wx(t)$ at time *t*. The goal of a BSS algorithm is to find a weight matrix *W* such that $\hat{s}(t)$ is a permutation of source signals *s*(*t*) up to a scaling factor. It is known that when *n* > *m* the principal component analysis (PCA) technique can be used to reduce the dimensionality of observations. For this reason, we only consider the case that *n* = *m* in this paper.

Since the pioneering work by Hyvärinen et al. [1], various separation algorithms have been proposed for different BSS subjects [2–6], for example, Oja et al. considered the nonnegative assumption and proposed some algorithms to separate these nonnegative sources [7–9]. A particular application of these algorithms is the blind separation of mixed images. More recently, much attention has been paid to BSS methods that make use of a priori information, such as sparse component analysis which works under the assumption that the sources can be represented by sparse signals. For the application of image separation, we here consider the a priori information that comes from the observation that most of neighbor pixels in a small patch are smooth. The local smoothness means little randomness, that is, the lower entropy values in the small patch compared to that of the image space. As stated in [10] that the smooth degree of any linear mixture of the source images is between the greatest smooth degree and the smallest smooth degree of the source images, we can formulate a proper entropy like function so that the source images would have the lowest entropy and their mixtures would have higher entropy values. By taking the entropy like function as the objective function, the global search technique is used to obtain the lowest entropy values of image signal, that is, the source images. The two-dimensional matrix formats will be treated for utilizing the full information carried by images. The result of experiment demonstrated that our method provides a good separation performance even for rich texture images. In [11], by using SVD technique, the mixed images are decomposed into three parts and the global stochastic optimization technique is used to recover the source images by searching for the lowest entropy values of images. Although Guo and Garland's algorithm has achieved a better performance compared to the conventional ICA method, however, it will cause large memory requirements. In other words, their algorithm is infeasible on most of computers. In [10], the separation performance is low in separating texture images.

## 2. Materials and Methods

### 2.1. Problem Statement

**Figure 1:**
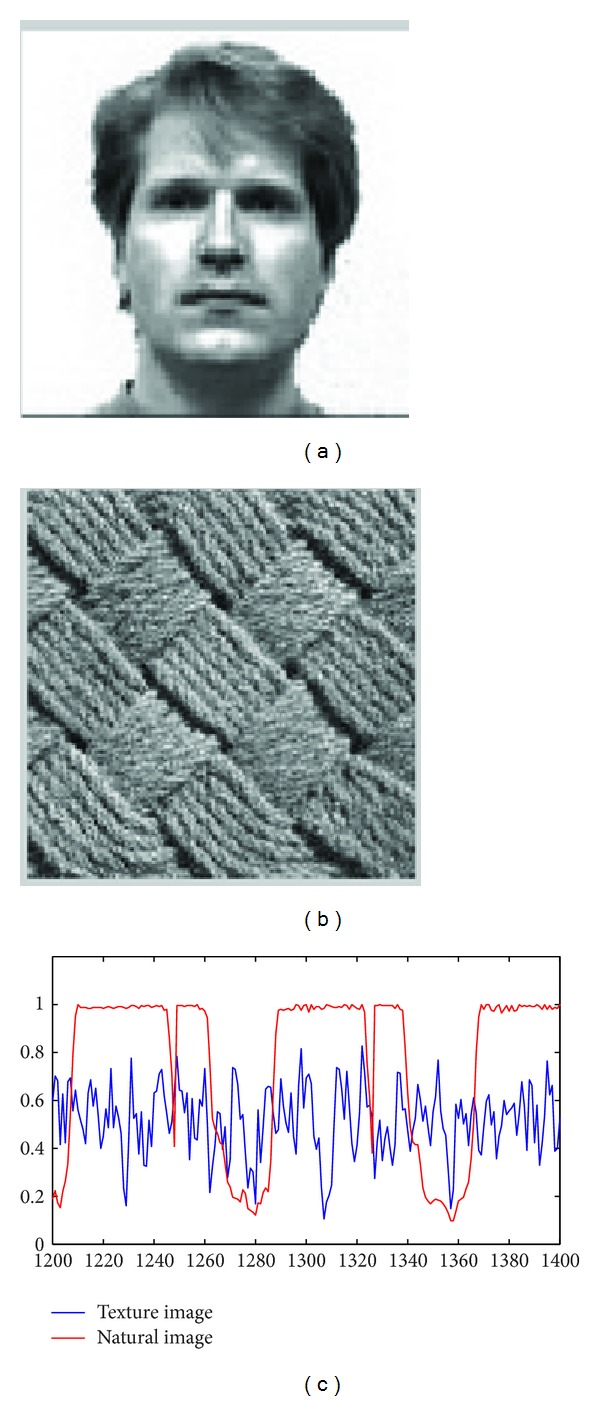
(a) Natural image; (b) textural image; (c) the segments of textural image and natural image.

For natural image signal, the neighbor pixels in a small patch always present strongly smooth property, which means that images are locally smooth (see [10] for more details). However, the smooth property is inapplicable to those images with rich texture. For example, the textural image is coarser according to the six features of image proposed by Tamura et al. To evaluate the coarseness of natural image and textural image, the following coarseness measure is used, which is defined in [12],

(2) $$\begin{array}{c} F_{crs}=\frac{1}{m\times n}\overset{m}{\underset{i}{\sum }}\;\overset{n}{\underset{j}{\sum }}S_{\text{best}}\left(i,j\right), \end{array}$$

where *m* and *n* are the effective width and height of the picture, respectively. The coarseness of natural image (Figure 1(a)) and textural image (Figure 1(b)) is, respectively, 0.9754 and 30.9368. Two segments of the natural image and the textual image, which are transformed to a vector in row-wise order, respectively, are drawn in Figure 1(c). Both images are of unit variance. From this key observation, the textural image is more random than natural image.

**Figure 2:**
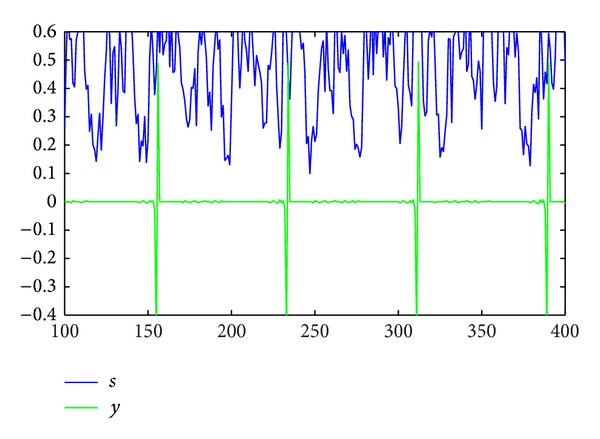
Comparison of *s* and *y*.

Motivated by the special phenomenon depicted in Figure 1(c), we can formulate (3) as follows:

(3) $$\begin{array}{c} y\left(k\right)=s\left(t\right)-s\left(t-k\right), \end{array}$$

where *k* represents a positive integer. The key point here is that *y*(*k*) has a large amount of columns in which only one element is not equal to zero. This phenomenon is shown in Figure 2.

By substituting (3) into (1), we can obtain the following equation:

(4) $$\begin{array}{c} z\left(k\right)=x\left(t\right)-x\left(t-k\right)=A\left(s\left(t\right)-s\left(t-k\right)\right). \end{array}$$

For image signals, some hypotheses are made naturally.

- Pixels are positive.
- There at least exist m vectors within the matrix *z*(*k*) which can construct the *n* × *m* matrix $\bar{A}$ which is a generalized permutation of the mixing matrix A up to a scaling factor.

For image signals, the hypothesis *A* is very normal, and hypothesis B can be met by modulating parameter *k*. Since *y*(*k*) has a large amount of columns that only one element is not equal to zero, the matrix *z*(*k*) would contain some columns which can form the matrix $\bar{A}\in R^{n\times m}$, which is a permutation of *A* up to a scaling factor. Namely, the matrix $\bar{A}$ is equivalent to the mixing matrix *A* by using normalization. Thus, the new matrix can be achieved as follows:

(5) $$\begin{array}{c} \bar{s}={\bar{A}}^{-1}x, \end{array}$$

where (−1) represents inverse operator. As stated above, the entropies of pure images would be lower than those of the mixed images. We take the form of (6) to minimize an entropy like function to approximate the entropy of $\bar{s}$ [11, 13]:

(6) $$\begin{array}{c} E=-\underset{i}{\sum }\;\underset{j}{\sum }p_{ij}\ln p_{ij}, \end{array}$$

where *p*_*ij*_ = (|*x*(*i* + 1, *j* + 1) + *x*(*i*, *j*) − *x*(*i*, *j* + 1) − *x*(*i* + 1, *j*)|)/(∑|*x*(*i* + 1, *j* + 1) + *x*(*i*, *j*) − *x*(*i*, *j* + 1) − *x*(*i* + 1, *j*)|). The probability distribution *p*_*ij*_ is the second derivative of the data [11]. In other words, the entropy like function *E* estimates the smoothness of the images. The pure source images can be obtained by solving the following minimization problem:

(7) $$\begin{array}{c} \min F_{\text{obj}}=E+\max\left\{p\times \left(e^{q/(1-\text{corr})}-1\right)\right\}, \end{array}$$

where corr denotes the 2D correlation coefficient between any two matrices and the parameter *q* = 0.0002 and *p* = 100000, empirically. Thus, we can take (7) as the objective function of global search algorithm.

### 2.2. The Standard Global Search Algorithm

In this paper, we use global search algorithm, such as Genetic Algorithm (GA) which is a useful solution to optimize and search problems, to achieve pure images. Generally, GA, one of the popular global stochastic optimization techniques, has been used to separate blind sources. This algorithm belongs to the larger class of evolutionary algorithms which are stemmed from the natural genetics and biological evolutionary process. The GA evaluates a population and generates a new one iteratively, with each successive population referred to as a generation. Given the current generation at iteration *t*, *G*(*t*), the GA generates a new generation *G*(*t* + 1), based on the previous generation, applying a set of genetic operations. The GA uses three basic operators to manipulate the genetic composition of a population: selection, crossover, and mutation [14]. Selection process determines the individuals for reproduction and the number of offspring that an individual can produce. Generally, we select ninety percent of individuals to produce new individuals and keep ten percent of individuals which have minimum values. During the selection process, each individual of current population is assigned a fitness value derived from the corresponding objective function value. Then, the selection algorithm selects individuals for reproducing on the basis of their relative fitness values. In our method, the fitness values are calculated using linear ranking method with pressure two which can prevent premature convergence [12]. The fitness of *i*th individual in the population is defined as follows:

(8) $$\begin{array}{c} F\left(x_{i}\right)=2-\mathrm{Max}+\;2\left(\mathrm{Max}-1\right)\frac{x_{i}-1}{p-1}, \end{array}$$

where Max is always chosen in [1.1, 2], which is used to determine the selective pressure such that no individuals generate an excessive number of offspring. And *x*_*i*_ is the position of the *i*th individuals in the reordered population based on their corresponding objective function values. The crossover operator mixes the genes of two chromosomes selected in the phase of reproduction, in order to combine the features, especially the positive ones of them. In the proposed algorithm, the simplest form of crossover is used, that is, one-point crossover.

### 2.3. The Proposed Algorithm

Based on the state above, we first utilize (3) to construct the new dataset *t*. Then we discard those columns, whose all elements are equal to zero and change those columns, whose all elements are negative values, to positive value by multiplying by −1. After that, reindex the vectors in *z*(*k*). In order to obtain a large amount of information of mixed image, the value of *k* should not be large. Those columns, whose elements are all equivalent to zero, should be discarded from *z*(*k*). At the same time, those columns, whose elements are negative, should be changed to nonnegative value by multiplying by −1. Then we will call GA twice. In the first time, the first pure image would be obtained by minimizing the objective function using GA. That is to say, we can find m vectors, which can form the matrix $\bar{A}$, from *z*(*k*). It means that the first pure image can be obtained when the correlation coefficient is the minimum. The first pure image would be saved and the GA is called again. Then we compare all separated images with the first pure image. In the case of only one separated image highly related with the pure image and the others which are mostly uncorrelated with the pure image, we can select a matrix of $\bar{A}$ to separate mixed images in each iteration. At the end of iteration, we can achieve a best matrix from $\bar{A}$.

The blind separation algorithm based on GA can be summarized as follows.

1. Form dataset *z*(*k*) by computing *z*(*k*) = *x*(*t*) − *x*(*x* − *k*).
2. Change *z*(*k*) to be nonnegative and discard those columns whose all elements are equal to zero.
3. Reindex the vectors in *z*(*k*).
4. Use GA to get the first pure image and save this image as reference image.
5. Call GA and compare all separated images with the first pure image by computing the correlation iteratively. Thus, a best separated matrix can be achieved.
6. Estimate $\bar{S}(t)$ by computing  (5).

## 3. Results

### 3.1. Separation of Texture Image

**Figure 3:**
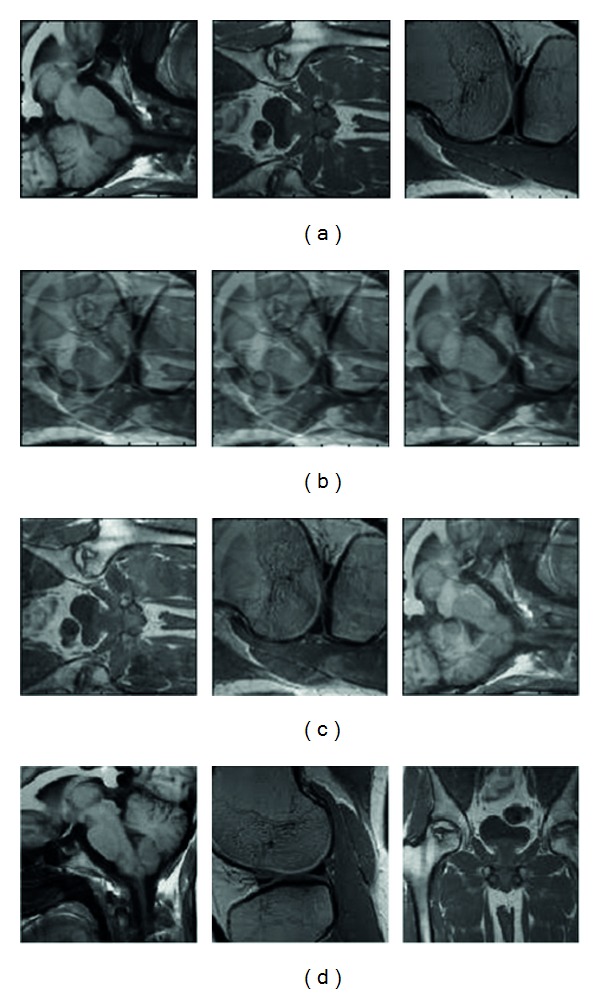
Results of standard ICA and the proposed method on 3 correlated MRIs with noise variance 0.01. (a) Source images; (b) mixed sources; (c) separation results using ICA method; (d) separation result using the proposed method.

In order to evaluate the proposed method, we tested MR images [15]. The digital imaging and communications in medicine (DICOM) standard was created by the National Electrical Manufacturers Association (NEMA) to aid the distribution and viewing of medical images, such as MR scans, and ultrasound. For this experiment, we have collected 3 MR scans whose correlation coefficients are between 0.6 and 0.8. The separated results are presented as follows. Figures 3(a), 3(b), and 3(c) illustrate the results of proposed algorithm. For simplicity, we here only plot the separated images with the variance *σ* ^2^ = 0.01.

To evaluate the separation performance, the following performance index (PI) in [9] is used, which is defined by

(9) PI=∑i=1n(∑j=1n|Cij|max⁡k|Cik|−1)+∑j=1n(∑i=1n|Cij|max⁡k|Ckj|−1),

where $C_{ij}={\bar{A}}^{-1}A$ is the combination of the separating and mixing matrix. The PIindex is equal to zero if and only if the matrix $\bar{A}$ is a permutation of *A*. The comparisons of the two methods with different variances of zero mean Gaussian white noise averaging over 100 trials are listed in Table 1.

From Table 1, we can see that the proposed algorithm in a noise scenario is superior accuracy compared to the ICA method.

## 4. Discussion

**Figure 4:**
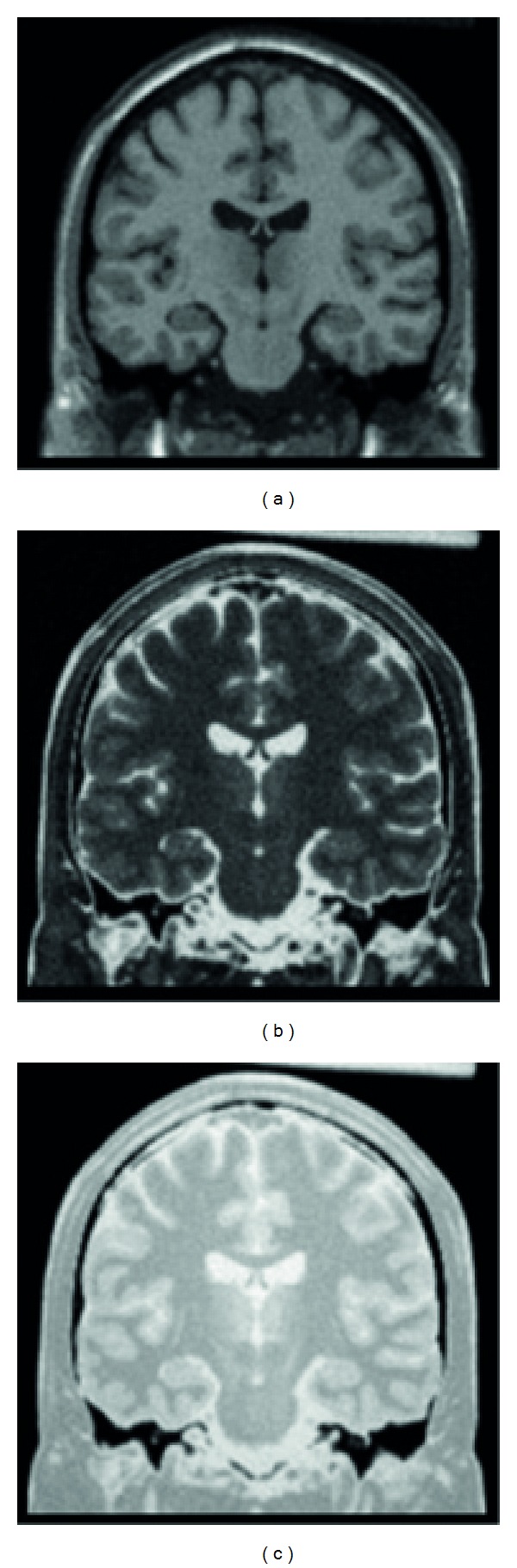
Simulated MR scans. (a) Spin-lattice; (b) spin-spin; (c) proton density.

The brain has a number of constituents in the context of a MRI scan of the brain, such as gray matter, white matter, cerebrospinal fluid (CSF) fat, muscle/skin, and glial matter. Now since each is unique, they would show unique characteristics under a magnetic field. However, while taking a scan, we get on MRI image of the entire brain. These scans can be considered as an equivalent to the mixtures of the blind source. The blind source separation technique can be used for this to separate out the various constituents such as gray matter, white matter, and CSF. These images of independent sources can be used for better diagnosis. The MR scans are from the McGill Simulated Brain Database as shown in Figure 4 [16].

**Figure 5:**
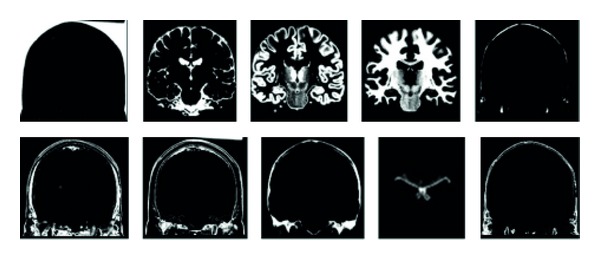
Ground truth images of different brain tissue substances.

Actually, the images [16] for these scans would be as shown in Figure 5.

Magnetic Resonance Imaging can give much better soft tissue contrast than that of CT for brain imaging, so MRI is superior to CT. It means that even small changes in the proton density and composition in the tissue are well represented by MRI. Some new methods and techniques can be used to improve scans obtained by MRI to improve diagnosis. Only in the past decade, various algorithms have been proposed to separate physiologically different components from EEG or MEG data [17, 18], financial data [19], and even in fMRI [20, 21]. However, for MRI, BSS-based methods have not gained much attention. Nakai et al. utilized ICA for the purpose of separating physiologically independent components from MRI scans [22]. They took MR images of 10 normal subjects, 3 subjects with brain tumor, and 1 subject with multiple sclerosis and performed ICA on the data. They reported success in improving contrast for gray and white matter, which was conducive to the diagnosis of brain tumor. The demyelination in multiple sclerosis cases was also enhanced in the images. The ICA method could potentially separate out all the tissues which had different relaxation characteristics according to their research result which shows much promise in biomedical domain. Take a set of MR frames as a single multispectral image, where each band is taken during a particular pulse sequence. Then ICA can be used on the data to separate out the physiologically independent components. Generally, a classifier such as the SVM would be used to improve the contrast of the separated components.

## 5. Conclusions

In this paper, a novel GA-based algorithm is proposed to separate MR images blindly by using smooth information in both noisy-free and noise scenarios. In order to take advantage of MR scans structure, we use an entropy like function to represent the local smooth property of near pixels. Let the entropy like function be the objective function, the GA is used for searching for the lowest entropy values. The performance of the proposed method is tested on NEMA MR image database. Simulations confirm the efficiency and effectiveness of the proposed algorithm. Because the standard GA method is sensitive to strong noise (see Table 1), further work is on the way to extend our method to the high noise scenario.

## Figures and Tables

**Table 1:** Separation performance with different variances of Gaussian white noise (average over 100 trials).

Algorithm	PI (*σ* ^2^ = 0)	PI (*σ* ^2^ = 0.01)	PI (*σ* ^2^ = 0.02)
Proposed method	2.3154*e* − 012	0.01324	1.5321
ICA method	4.2405	4.2305	4.7959
